# Proteogenomics in cerebrospinal fluid and plasma reveals new biological fingerprint of cerebral small vessel disease

**DOI:** 10.21203/rs.3.rs-4535534/v1

**Published:** 2024-07-02

**Authors:** Stephanie Debette, Ilana Caro, Daniel Western, Shinichi Namba, Na Sun, Shuji Kawaguchi, Yunye He, Masashi Fujita, Gennady Roshchupkin, Tim D’Aoust, Marie-Gabrielle Duperron, Murali Sargurupremraj, Ami Tsuchida, Masaru Koido, Marziehsadat Ahmadi, Chengran Yang, Jigyasha Timsina, Laura Ibanez, Koichi Matsuda, Yutaka Suzuki, Yoshiya Oda, Akinori Kanai, Pouria Jandaghi, Hans Markus Munter, Dan Auld, Iana Astafeva, Raquel Puerta, Jerome Rotter, Bruce Psaty, Joshua Bis, Will Longstreth, Thierry Couffinhal, Pablo Garcia-Gonzalez, Vanesa Pytel, Marta Marquié, Amanda Cano, Mercè Boada, Marc Joliot, Mark Lathrop, Quentin Le Grand, Lenore Launer, Joanna Wardlaw, Myriam Heiman, Agustin Ruiz, Paul Matthews, Sudha Seshadri, Myriam Fornage, Hieab Adams, Aniket Mishra, David-Alexandre Trégouët, Yukinori Okada, Manolis Kellis, Philip De Jager, Christophe Tzourio, Yoichiro Kamatani, Fumihiko Matsuda, Carlos Cruchaga

**Affiliations:** University of Bordeaux; University of Bordeaux; Department of Psychiatry, Washington University School of Medicine, St. Louis, MO, USA; Osaka University; MIT Computer Science and Artificial Intelligence Laboratory; Broad Institute of MIT and Harvard; Kyoto University Graduate School of Medicine; Graduate School of Frontier Sciences, The University of Tokyo; Columbia University Irving Medical Center; Erasmus Medical Center; Bordeaux Population Health, Inserm U1219, University of Bordeaux; University of Bordeaux, Inserm, Bordeaux Population Health Research Center, UMR 1219; University of Bordeaux, Inserm, Bordeaux Population Health Research Center, team VINTAGE, UMR 1219, F-33000 Bordeaux, France; Glenn Biggs Institute for Alzheimer’s & Neurodegenerative Diseases Unive; Graduate School of Frontier Sciences, The University of Tokyo; Victor Phillip Dahdaleh Institute of Genomic Medicine, McGill University; Washington University in St. Louis; Department of Psychiatry, Washington University School of Medicine, St. Louis, MO, USA; Washington University in St. Louis; Department of Computational Biology and Medical Sciences, Graduate school of Frontier Sciences, The University of Tokyo; The University of Tokyo; Graduate School of Medicine, The University of Tokyo; The University of Tokyo; McGill University; Victor Phillip Dahdaleh Institute of Genomic Medicine, McGill University; Bordeaux Population Health, Inserm U1219, University of Bordeaux; Institute of Neurodegenerative Diseases; Ace Alzheimer Center Barcelona; The Lundquist Institute for Biomedical Innovation at Harbor-UCLA Medical Center; Cardiovascular Health Research Unit; University of Washington; University of Washington; University of Bordeaux, The clinical unit of Exploration, Prevention and Care Center for Atherosclerosis (CEPTA), CHUB, Inserm U1034; Ace Alzheimer Center Barcelona; Ace Alzheimer Center Barcelona, Universitat Internacional de Catalunya; CIBERNED, Network Center for Biomedical Research in Neurodegenerative Diseases, National Institute of Health Carlos III; ACE Alzheimer Center Barcelona; Ace Alzheimer Center Barcelona; Universitat Internacional de Catalunya; GIN, IMN/UMR5293 UB/CNRS/CEA; Department of Human Genetics, McGill University, 1205 Dr Penfield Avenue, Montreal, QC, H3A 1B1, Canada; University of Bordeaux, Inserm, Bordeaux Population Health Research Center, UMR 1219; National Institute on Aging, National Institutes of Health; University of Edinburgh; Massachusetts Institute of Technology; Glenn Biggs Institute for Alzheimer’s and Neurodegenerative Diseases, University of Texas Health Sciences Center; Ace Alzheimer Center Barcelona, Universitat Internacional de Catalunya;CIBERN; UK Dementia Research Institute Centre at Imperial College London; University of Texas Health Science Center; 1. Institute of Molecular Medicine, McGovern Medical School, The University of Texas Health Science Center 2. Human Genetics Center, Department of Epidemiology, School of Public Health; Department of Human Genetics, Radboud University Medical Center; Latin American Brain Health (BrainLat), Universidad Adolfo Ibáñez; University of Bordeaux; INSERM; Department of Genome Informatics, Graduate School of Medicine, The Univ. of Tokyo; Department of Statistical Genetics, Osaka Univ. Graduate School of Medicine; Laboratory for Systems Genetic, RIKEN; MIT; Columbia University Irving Medical Center; The University of Tokyo; Kyoto University Graduate School of Medicine; Washington University

## Abstract

Cerebral small vessel disease (cSVD) is a leading cause of stroke and dementia with no specific mechanism-based treatment. We used Mendelian randomization to combine a unique cerebrospinal fluid (CSF) and plasma pQTL resource with the latest European-ancestry GWAS of MRI-markers of cSVD (white matter hyperintensities, perivascular spaces). We describe a new biological fingerprint of 49 protein-cSVD associations, predominantly in the CSF. We implemented a multipronged follow-up, across fluids, platforms, and ancestries (Europeans and East-Asian), including testing associations of direct plasma protein measurements with MRI-cSVD. We highlight 16 proteins robustly associated in both CSF and plasma, with 24/4 proteins identified in CSF/plasma only. cSVD-proteins were enriched in extracellular matrix and immune response pathways, and in genes enriched in microglia and specific microglial states (integration with single-nucleus RNA sequencing). Immune-related proteins were associated with MRI-cSVD already at age twenty. Half of cSVD-proteins were associated with stroke, dementia, or both, and seven cSVD-proteins are targets for known drugs (used for other indications in directions compatible with beneficial therapeutic effects. This first cSVD proteogenomic signature opens new avenues for biomarker and therapeutic developments.

## Introduction

Characterized by changes in the structure and function of small brain vessels, cerebral small vessel disease (cSVD) is a leading cause of ischemic and hemorrhagic stroke, cognitive decline and dementia. cSVD is extremely common with increasing age and most often covert, namely detectable on brain imaging in the absence of clinical symptoms. Covert cSVD portends a considerably increased risk of stroke and dementia, thus represents a major target to prevent these disabling conditions and promote healthier brain aging^1^. The most common and heritable MRI-markers of cSVD (MRI-cSVD) are white matter hyperintensities of presumed vascular origin (WMH) and perivascular spaces (PVS)^2^.

Hypertension is the strongest known risk factor for cSVD, representing a major target for prevention^1^. However, vascular risk factors explain only a small fraction of MRI-cSVD variability in older age^3^, and drugs specifically targeting pathological processes underlying cSVD are lacking. Genomics can provide a strong foundation for mechanistic studies and drug target discovery^4^. Recent genetic studies have identified > 70 genetic risk loci associated with cSVD^5,6^; however, causal genes and underlying molecular pathways remain poorly understood.

As disease occurrence reflects the complex interplay of factors beyond DNA sequence, there is growing interest in identifying circulating biomarkers, such as proteins, capturing these downstream factors, to enhance our understanding of the underlying biology, accelerate omics-driven drug discovery, and potentially generate circulating biomarkers for clinical use^7^. While large-scale proteomic investigations have recently been conducted for stroke and dementia, with promising findings,^7–13^ studies on proteomics of cSVD have been conducted on limited sets of proteins, in small studies of European ancestry (N < 5,000), and in plasma only^14–18^. We hypothesize that, while plasma may enable easy-access biomarker measurements, CSF, the fluid circulating in perivascular spaces, could reveal a more accurate biological fingerprint of cSVD.

Here we used two-sample Mendelian randomization (2SMR), leveraging large proteomic and genomic resources, to investigate the relation of circulating protein levels in CSF and plasma with WMH and PVS burden and to explore its causal relation and directionality. We further used a multipronged approach for the follow-up of identified associations in independent samples, across fluids, proteomics platforms, ancestries and the lifespan, using both 2SMR and individual-level data. We also explored the ability of proteogenomics to predict extensive cSVD and tested the relation of cSVD-associated proteins with risk of stroke and Alzheimer’s disease (AD). Using single-cell sequencing resources we deciphered cell-types and pathways involved. Finally, we combined our results with pharmacological databases for proteomics-driven drug discovery.

## Results

**Figure 1:**
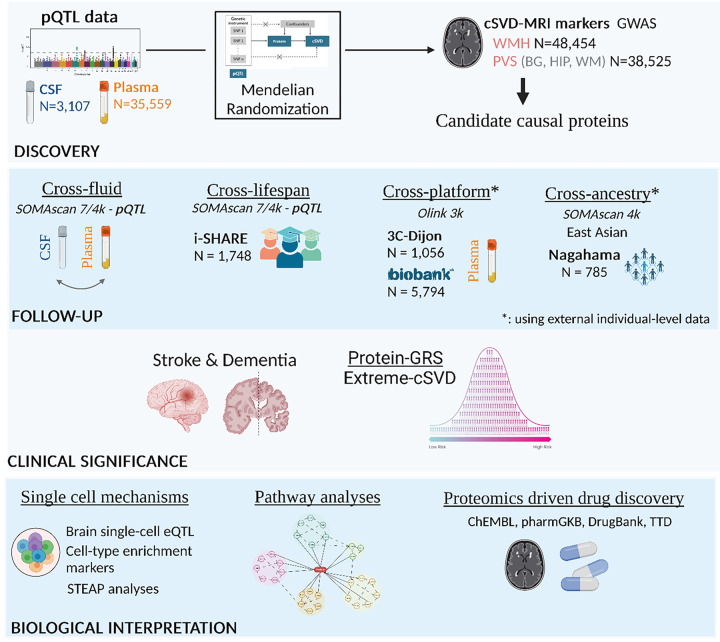
Summary of the analysis plan. pQTL: protein quantitative trait loci, CSF: Cerebrospinal fluid, WMH: White matter hyperintensities, PVS: Perivascular Spaces burden, BG: basal ganglia, HIP: hippocampus, WM: white matter. # Cross-platform follow-up analyses have been conducted using a meta-analysis of 3C and UK Biobank

The study design is summarized in Fig. 1.

### Discovery of protein-cSVD associations

We used 2SMR to test associations of circulating CSF and plasma protein levels with MRI-cSVD. We leveraged summary statistics of large protein quantitative trait loci (pQTL) resources in European-ancestry participants from CSF^12^ (N = 3,107; aptamer-based Somascan 7k assay) and plasma^19^ (N = 35,559; Somascan 5K), as well as the largest published GWAS of WMH volume (N = 48,454, mean age 66.0 years)^20^ and PVS burden (N up to 40,095, mean age 66.3 years)^5^. PVS were studied in three sublocations, white matter (WM), basal ganglia (BG) and hippocampus (HIP), for which risk factors, including genetic, were shown to differ^5^. Cis- and trans-genetic instruments could be derived for 1,121 CSF and 1,731 plasma proteins. (Methods)

**Figure 2:**
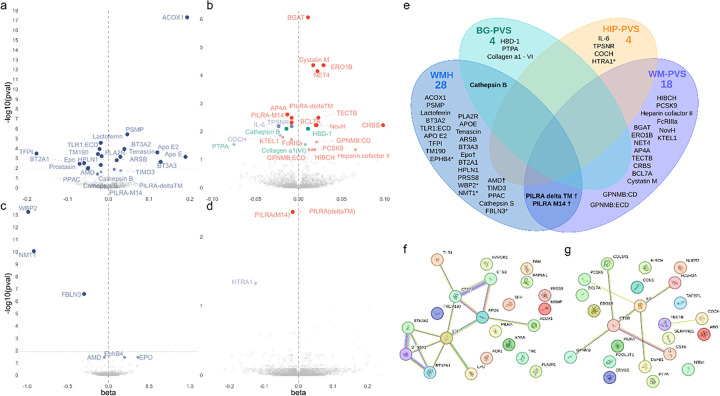
Discovery protein-cSVD associations in CSF and plasma using cis-pQTL mendelian randomization. A. Volcano plots of proteins associated with white matter hyperintensities (WMH) using cis-pQTL MR in CSF. B. Volcano plots of proteins associated with perivascular spaces burden (PVS) using cis-pQTL MR in CSF. C. Volcano plots of proteins associated with WMH using cis-pQTL MR in plasma. D. Volcano plots of proteins associated with PVS using cis-pQTL MR in plasma. Each dot represents the MR results for proteins. Each dot represents the MR results for proteins. FDR-corrected p-values are represented in this graph. Represented proteins are significantly associated with MRI-marker at p_FDR_ (Benjamini-Hochberg false discovery rate threshold) < 0.05. The dotted line in each volcano plot represents the corrected threshold after additionally correcting for the number of phenotypes tested (p<0.0125). E. Venn diagram of identified causal proteins associated with MRI-cSVD. * proteins identified in plasma; † proteins associated in both plasma and CSF; other proteins are associated in CSF. F. String plot of proteins associated with WMH. G. String plot of proteins associated with PVS (WM, BG and HIP). Network nodes represent proteins: colored nodes query proteins and first shell of interactors. Edges represent protein-protein associations. Cyan and pink edges are known interactions, cyan: from curated databases, and pink: experimentally determined. Green and blue edges correspond to predicted interactions. Green: gene neighborhood, and blue: gene co-occurrence. Purple corresponds to protein homology, yellow to text mining and black to co-expression.

Focusing our primary analyses on *cis*-pQTLs (**Supplementary Table 1**), we identified 46 of 1,121 CSF proteins associated with at least one MRI-cSVD (p_FDR_<0.05): 24 with WMH, and 25 with PVS (18 WM-PVS, 4 BG-PVS, and 3 HIP-PVS, Fig. 2A–B, **Supplementary Table 2–3)**. In parallel we identified 9 of 1,731 plasma proteins associated with MRI-cSVD (p_FDR_<0.05): 6 with WMH and 3 with PVS (2 WM-PVS, 1 HIP-PVS). Of these, 4 were also significantly associated with MRI-cSVD in CSF (AMD, EPO [WMH], PILRA-M14 and PILRA-deltaTM [WM-PVS], Fig. 2C–D, **Supplementary Tables 4–5)**. For pQTL with multiple instruments (42 proteins), associations were robust to sensitivity analyses (MR-Egger, weighted median and MR-PRESSO); for single-instrument pQTL (14 proteins), there was no evidence of colocalization for two proteins, ACOX1 and WBP2 with PP4 < 0.7 & PP3 > 0.7, which were removed from subsequent analyses (**Supplementary Tables 3 and 5**). None of the single variant pQTL were non-synonymous variants, which could have resulted in structural changes at the aptamer protein binding site and thus biased its measurement (**Supplementary Table 6**). Bidirectional MR ruled out reverse causation, except for an association of genetically determined larger WM-PVS burden with higher PCSK9 CSF levels (p_FDR_=0.011, **Supplementary Table 3**). In total, 49 proteins were associated robustly with MRI-cSVD in CSF (41), plasma (4), or both (4), including three associated with both WMH and PVS: CTSB (Cathepsin B), a lysosomal protease involved in extracellular matrix (ECM) degradation, and two soluble isoforms deltaTM and M14 of PILRA (Paired Immunoglobin Like Type 2 Receptor Alpha), a microglial immunoreceptor.

In secondary analyses including both *cis*- and *trans*-pQTLs, we found 340 proteins associated with at least one MRI-cSVD in CSF or plasma (p_FDR_<0.05), of which 176 were driven by two trans-hotspots at *APOE* (147 proteins) and chr16q24 (29 proteins). Although most protein-cSVD associations revealed novel pathways not previously identified, some relate to previous cSVD GWAS findings. Two *cis*-pQTL were associated with WMH volume at genome-wide significance, for FBLN3 (encoded by *EFEMP1*) at chr2p16 and NMT1 (*NMT1*) at chr17q21. Additionally, HTRA1, of which lower genetically determined plasma levels were associated with extensive HIP-PVS, is encoded by a gene harboring both rare mutations causing monogenic cSVD^21^ and common variants associated with small vessel stroke and suggestively WMH^20,22^. From secondary analyses, eight *trans*-pQTL for 29 proteins at the chr16q24 hotspot were associated at genome-wide significance with WMH. The APOE hotspot included four proteins encoded by genes in genome-wide or gene-wide significant risk loci for WMH^20^ and extreme-cSVD^23^ (*APOE, MRPL38, SULT1B1*, and *MSRA*; **Supplementary Tables 6–9, Extended Data** Fig. 1).

To assess the independence of observed associations, we used LD-score regression (LDSC)^24^ to quantify the genetic correlation between protein levels. Only one genetic correlation was significant after multiple testing correction (EPHB4 with PILRA-M14 in plasma at p < 5×10^− 5^, Methods**, Extended Data** Fig. 2). Several protein-protein interactions were identified using the STRING database (Fig. 2F).

### Follow-up of significant protein-cSVD associations

**Figure 3:**
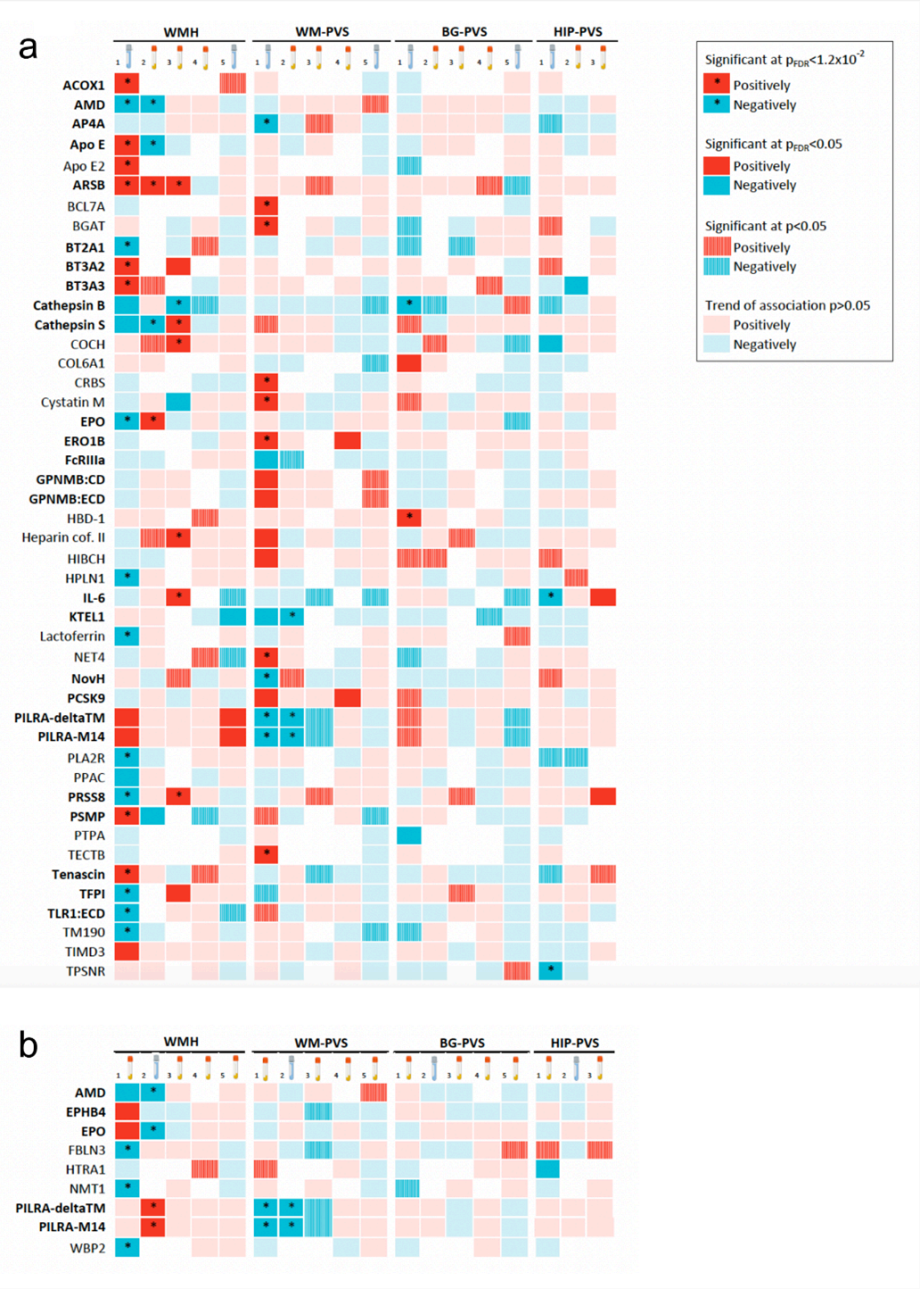
Summary of proteomics follow-up (discovery, cross-fluid, cross-platform, cross-ancestry and lifespan) A. Heatmap of proteomic findings using CSF discovery analysis. B. Heatmap of proteomic findings using plasma discovery analysis. 1. Discovery Mendelian randomization using cis-pQTL from CSF (A) and plasma (B). 2. Cross-fluid follow-up Mendelian randomization using cis-pQTL from plasma (A) and CSF (B). 3. Cross-platform follow-up using plasma individual-level data measured with Olink in independent samples (3C, UKB). 4. Cross-ancestry follow-up using plasma individual-level data measured with Somascan in an independent sample (Nagahama). 5. Lifespan follow-up Mendelian randomization using cis-pQTL from CSF (A) and plasma (B). Dark squares correspond to significant results after FDR correction (p_FDR_<0.05). * corresponds to significant associations after correction for the 4 phenotypes tested (p_FDR_<0.0125). Hatched squares correspond to p<0.05 results. Red squares correspond to a positive association and blue to negative association. Proteins missing for one of the follow-up analyses are represented with a white square. # results of the analysis of 3C only. Proteins in bold are those showing at least one nominally significant association (p<0.05) in follow-up analyses, with the same MRI-cSVD marker as in the discovery.

We used a multi-pronged approach to follow-up protein-cSVD associations based on *cis*-pQTL with significant MR results and colocalization evidence, across fluids, platforms, and ancestries (Figs. 1 and 3).

First, using 2SMR, we tested whether cSVD-proteins associations observed in CSF showed some indication of association in plasma, and vice-versa, with a less stringent multiple testing correction than in the discovery analysis, considering significant associations in the original fluid only. Thirty-seven cSVD-associated CSF proteins had plasma pQTL available. Nine of these (24%) were associated with the same MRI-cSVD phenotype in plasma at p_FDR_<0.05 (APOE, ARSB, EPO, AMD, CTSS, PSMP with WMH, PILRA-M14, PILRA-deltaTM, KTEL1 with WM-PVS, Methods, Fig. 3A and 6, **Supplementary Table 10**). Six cSVD-associated plasma proteins had CSF cis-pQTL available. Four of these (67%) were associated with the same MRI-cSVD phenotype in CSF at p_FDR_<0.05 (AMD, EPO with WMH and PILRA-M14, PILRA-deltaTM with WM-PVS, Fig. 3B and 6, **Supplementary Table 11**). Directions of association were mostly concordant except for EPO, APOE and PSMP, which showed opposite direction of association in CSF and plasma, in line with previous observations^12^ and highlighting the importance of studying multiple tissues to capture the complexity of underlying biology.

Second, a cross-platform follow-up was performed by testing the association with MRI-cSVD of plasma protein levels measured on the Olink Explore-3072 platform in two independent population-based studies, 3C-Dijon (N = 1,056; mean age 72.5 years) and UK Biobank (N = 5,494; mean age 63.5 years, **Supplementary Table 12**). Twenty-nine of the 49 cSVD-associated proteins (59%) were available; 26 were used after quality control and their plasma level was tested against WMH volume and PVS burden using linear regression followed by inverse variance weighted meta-analysis (N = 6,550). Of these, 7 proteins (27%, all identified in CSF 2SMR), showed association with the same MRI-cSVD marker at p_FDR_<0.05 (ARSB, PRSS8, CTSS, CTSB, TFPI and BT3A2 with WMH, IL-6 with HIP-PVS, Figs. 3 and 6, **Supplementary Tables 13–14**). Directionality of association with MRI-cSVD was inconsistent between CSF pQTL and plasma protein levels for PRSS8, TFPI, IL-6, and CTSS. Inter-platform correlations for these proteins between Somascan and Olink were moderate to good in plasma and CSF respectively (**Supplementary Table 15**^25^); however correlations were not available between plasma and CSF.

Third, we conducted a cross-ancestry exploratory follow-up, testing associations of MRI-cSVD with plasma protein levels measured on the Somascan 4K platform in the Japanese population-based Nagahama study (N = 785; mean age 68 years). Thirty-eight of the 49 cSVD-associated proteins (77%) were available and their plasma level was tested against WMH volume and extensive PVS burden. Two proteins (both identified in CSF 2SMR in Europeans) were associated at p_FDR_<0.05 with the same MRI-marker (WM-PVS), with consistent directionality (ERO1B and PCSK9); given the small sample size we also considered nominally significant associations, observed for four additional proteins, with WMH (BT2A1, CTSB, TNC, PSMP, Figs. 3 and 6, **Supplementary Table 16**).

Fourth, we took an exploratory lifespan approach by testing the relation of cSVD-associated proteins with MRI-cSVD in young adults (i-Share study, N = 1,748; mean age 22.1 years). Here we used 2SMR with the same *cis*-pQTL as for discovery analyses. Consistent with findings in older adults, higher genetically determined CSF levels of PILRA-M14, PILRA-deltaTM were associated with larger WMH volume at p_FDR_<0.05. In addition, higher genetically determined CSF protein levels of GPNMB:CD and GPNMB:ECD (cellular and extracellular domain of a transmembrane glycoprotein upregulated upon tissue damage and inflammation) and TLR1:ECD (extracellular domain of toll-like receptor 1, which plays a fundamental role in activation of innate immunity) were associated with BG-PVS and WMH volume respectively at p < 0.05, in a direction consistent with older adults (Fig. 3, **Supplementary Table 17)**.

Overall, of 49 cSVD-associated proteins (**Supplementary Table 18**, Fig. 6), (i) 16 CSF proteins showed associations with the same MRI-cSVD marker in plasma in at least one analysis (pQTL or direct protein measurement) at p_FDR_<0.05, with consistent directionality in 63%; (ii) 24 CSF proteins were not associated with the same MRI-marker in plasma (p ≥ 0.05) and may be considered as CSF-specific; (iii) 4 proteins were identified in plasma pQTL analyses only, with non-significant follow-up in association with direct plasma protein measurements; (iv) 5 proteins had no follow-up available apart from the lifespan exploration; (iii) and 6 proteins had evidence for lifespan effects at p < 0.05 (2 at p_FDR_<0.05).

### Predictive performance of protein genetic risk scores (GRS)

We assessed the ability of selected *cis*-based protein-GRS to predict a composite extreme-cSVD phenotype (extensive WMH volume ± lacunes vs. minimal WMH volume without lacunes) in the 3C-Dijon cohort, benchmarking it against a previously validated WMH-GRS^20^ (Methods). Using the WMH-GRS only, we achieved an AUC of 0.568 (95% Bootstrap CI 0.501–0.634). Adding any of the four selected protein GRS slightly improved the AUC, while adding them all achieved a maximum improvement of + 0.04 (AUC = 0.608; 95% CI [0.544–0.672], **Extended Data** Fig. 4 **Supplementary Table 19**).

### Clinical significance

**Figure 4:**
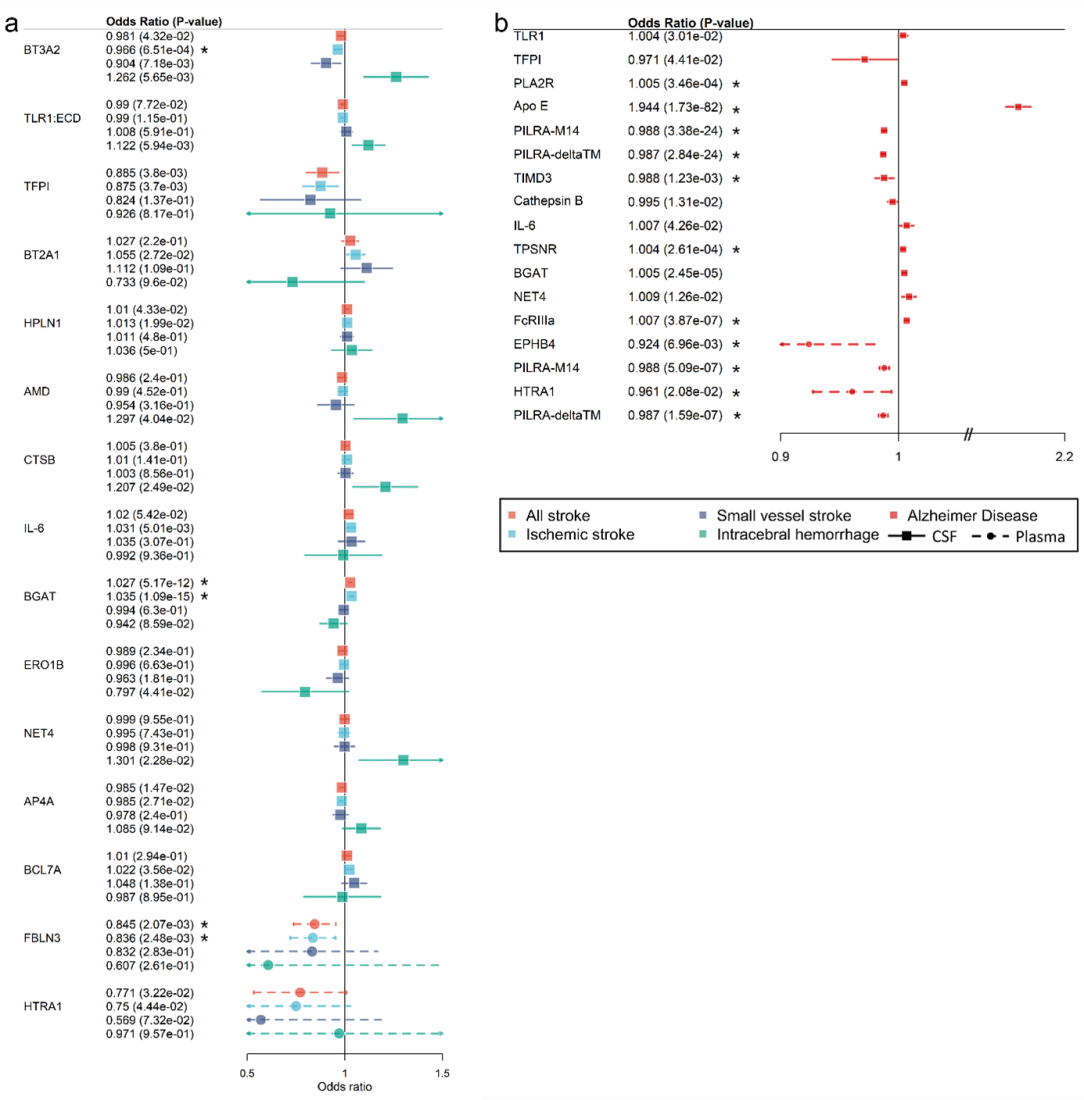
Clinical significance of protein-cSVD findings in CSF and plasma. A. Forest plot of protein-cSVD associations with stroke and its subtypes (ischemic stroke, small vessel stroke and intracerebral hemorrhage). B. Forest plot of protein-cSVD association with Alzheimer’s disease. All proteins associated with MRI-cSVD identified in the discovery analysis in CSF and plasma were used for this analysis. Full lines represent proteins measured in CSF. Dotted lines represent proteins measured in plasma. Proteins significant at least at p<0.05 for at least one of the outcomes tested are represented (for stroke, associations with all (sub)types are represented when one or more was significant). * Results significant after multiple testing correction (p_FDR_<0.05)

We explored the relation of the 49 cSVD-associated proteins with stroke (any, ischemic, and small vessel stroke; intracerebral hemorrhage) and AD (Methods). We leveraged the aforementioned CSF and plasma pQTL, as well as European-ancestry summary statistics of GWAS for stroke and its subtypes (N ≤ 73,652 cases) and AD (N = 71,880 cases). Twenty-four proteins (49%) showed associations with at least one clinical outcome at p < 0.05 (Figs. 4 and 6). At p_FDR_<0.05, eight CSF proteins (APOE, PILRA-M14, PILRA-deltaTM, FcRIIIa, BGAT, PLA2R, TIMD3 and TPSNR) and four plasma proteins (EphB4, HTRA1, PILRA-M14, PILRA-deltaTM) were significantly associated with AD, while one CSF protein (BGAT, measuring histo-blood group ABO system glycosyltransferase activity) and one plasma protein (FBLN3) were associated with any stroke and ischemic stroke (**Supplementary Tables 20–21)**. Nineteen of 49 proteins were available for partial follow-up in plasma using 2SMR in East-Asian participants in relation with ischemic and small vessel stroke, leveraging plasma pQTL from Biobank Japan (N = 2,886) and an East-Asian stroke GWAS meta-analysis (N ≤ 17,493). Overall, despite substantially smaller sample size for exposure and outcome in East-Asians, correlation of effect sizes was moderate to high (**Extended Data** Fig. 5). Higher plasma levels of NovH (encoded by *CCN3*), an ECM associated protein involved in cardiovascular development, were associated with increased risk of small vessel stroke at p_FDR_<0.05 (**Supplementary Table 22**).

### Biological interpretation

Using FUMA pathway enrichment analyses, cSVD-associated proteins overall were significantly enriched in proteins involved in proteoglycan binding and extracellular matrix (organization and collagen containing: CTSS, EFEMP1, HAPLN1, CTSB, HTRA1, NTN4, COL6A1, TNC, COCH, APOE, p_FDR_<0.05, **Supplementary Table 23A**). Among CSF proteins associated with cSVD, proteins involved in regulation of immune response signaling and activation of immune response were overrepresented (BT2A1, BT3A2, BT3A3, CTSB, CTSS, LTF, TLR1, HAVCR2, p_FDR_<0.05, **Supplementary Table 23B**).

To explore enrichment of observed protein-cSVD associations in particular cell-types we first conducted single-cell enrichment analyses using STEAP, leveraging multiple publicly available single-cell sequencing resources (Methods, Supplementary Table 24). Genes encoding several cSVD-associated proteins showed significant enrichment in microglia for several CSF proteins (BT2A1, BT3A2, BT3A3, CTSS, HIBCH) and in immune cells for plasma protein (EPO, **Supplementary Table 25, Extended data** Fig. 6). Next, we used unique resources of single nucleus RNA sequencing (snRNAseq) derived from up to 443 post-mortem brain samples (dorsolateral prefrontal cortex) from the ROSMAP older population-based cohort^26–29^. *In silico* sorting of human cortical tissue samples was used to derive vascular brain cells^27,28^. From these snRNAseq resources we could derive cell-type specific brain eQTLs for 19 and 10 genes encoding cSVD-associated proteins, in non-vascular and vascular cells respectively (Methods). Using MR, we found lower genetically determined expression levels of *TLR1* in oligodendrocytes (p_FDR_=2.24×10^− 4^) and *CTSS* in smooth muscle cells (p_FDR_=2.3×10^− 3^) to be associated with larger WMH volume, both consistent with directionality of associations in CSF (**Supplementary Table 26–27**). Higher genetically determined expression of *ABO* (encoding BGAT) in pericytes was protective for extensive WM-PVS (p_FDR_=2.3×10^− 3^, opposite direction compared to CSF). All three associations showed evidence for colocalization (PP.H4 > 0.7). Genes encoding cSVD-associated proteins showed distinct cerebrovascular cell-specific gene expression patterns (e.g. with *EFEMP1* expression dominating in a new subtype of perivascular fibroblasts) and we observed a non-significant trend towards an overall enrichment in pericytes (**Extended data Fig. 7**). We also tested enrichment of our genes of interest in different microglial states (Methods**, Extended data Fig. 7**), given the aforementioned results observed with STEAP, and observed significant enrichment in a microglial state type previously found to be itself enriched in processes such as ribosome biogenesis, amyloid fibril formation, and positive regulation of T-cell mediated immunity^29^.

### Proteomics-driven drug discovery

**Figure 5:**
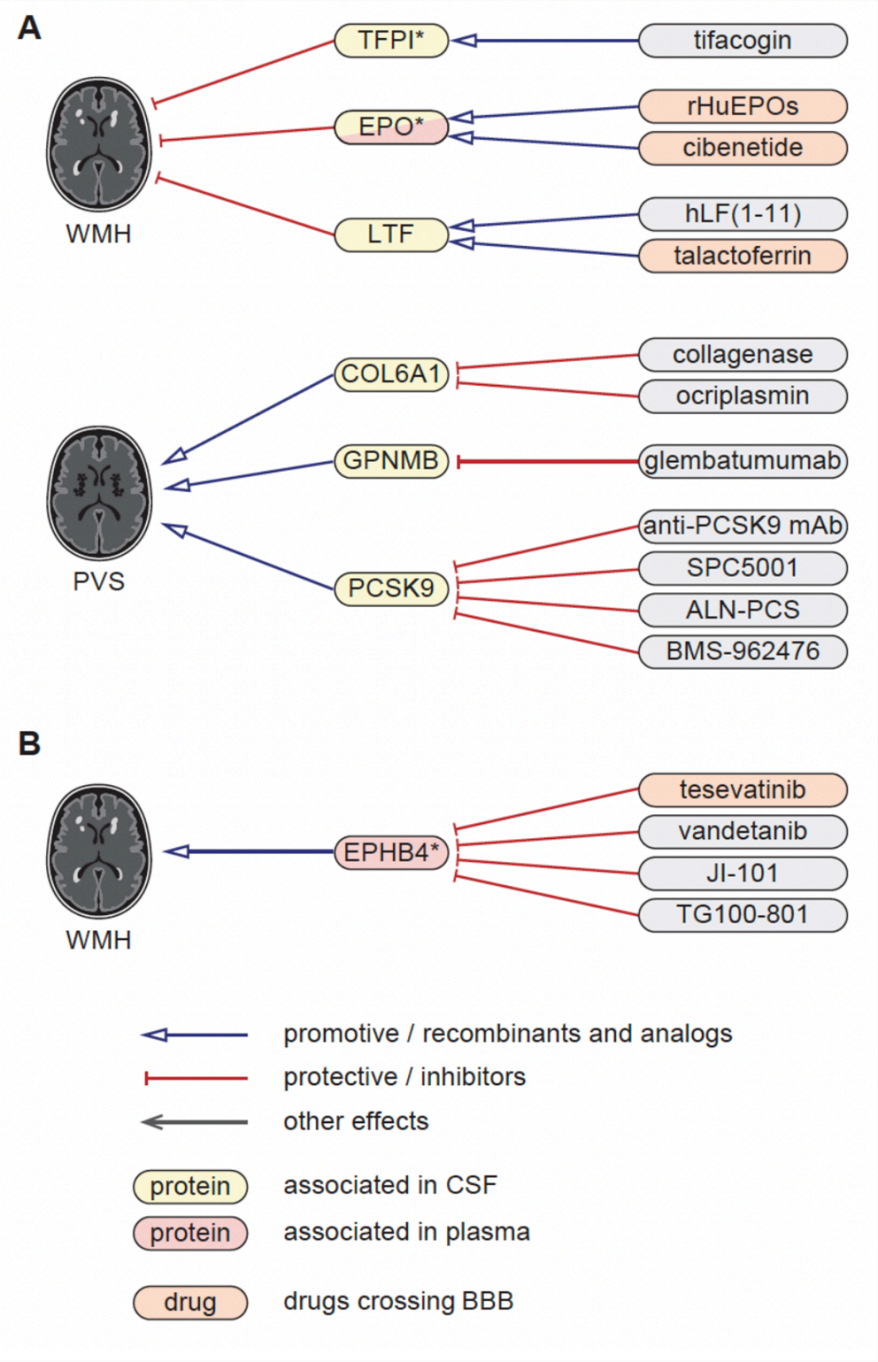
Proteomics-driven drug discovery. A. Drug-discovery analysis conducted using CSF protein-cSVD Mendelian randomization estimates for WMH and PVS findings. B. Drug-discovery analysis conducted using plasma protein-cSVD Mendelian randomization estimates for WMH. Proteins in yellow correspond to proteins associated with the MRI-cSVD marker in CSF and in red in plasma, in discovery analyses. * proteins with significant associations in at least one of the follow-up modalities (at p<0.05). Red arrows correspond to a protective effect of a protein on MRI-cSVD (reducing cSVD burden) or an inhibitor effect of a drug on the cSVD-associated protein; blue arrows correspond to a of deleterious effects of a protein on MRI-cSVD (promoting cSVD burden) or an analog effect of a drug on the cSVD-associated protein. Drugs in orange cross the blood brain barrier. CSF: cerebrospinal fluid, BBB: blood brain barrier.

We used MR estimates from the 49 CSF and plasma proteins with MRI-cSVD to support drug discovery. Using public drug databases (Methods), we curated drugs (commercialized for other indications or under investigation in clinical trials) targeting these proteins in a direction compatible with beneficial therapeutic effects against cSVD based on MR estimates. We identified such drugs for EPO, LTF, TFPI, and EPHB4 for WMH; COL6A1, GPNMB, PCSK9 for PVS, most of which were associated with MRI-cSVD in the CSF only, except EPHB4 (plasma), EPO and TFPI (CSF and plasma, Fig. 5, **Supplementary Table 28**). Some of these proteins have predicted or experimentally proven interactions with each other (Fig. 2E–F), suggesting that identified drugs may impact related pathways. Of note, drugs targeting EPO and LTF as agonists and EPHB4 as inhibitors cross the blood-brain barrier (**Supplementary Table 28**).

**Figure 6:**
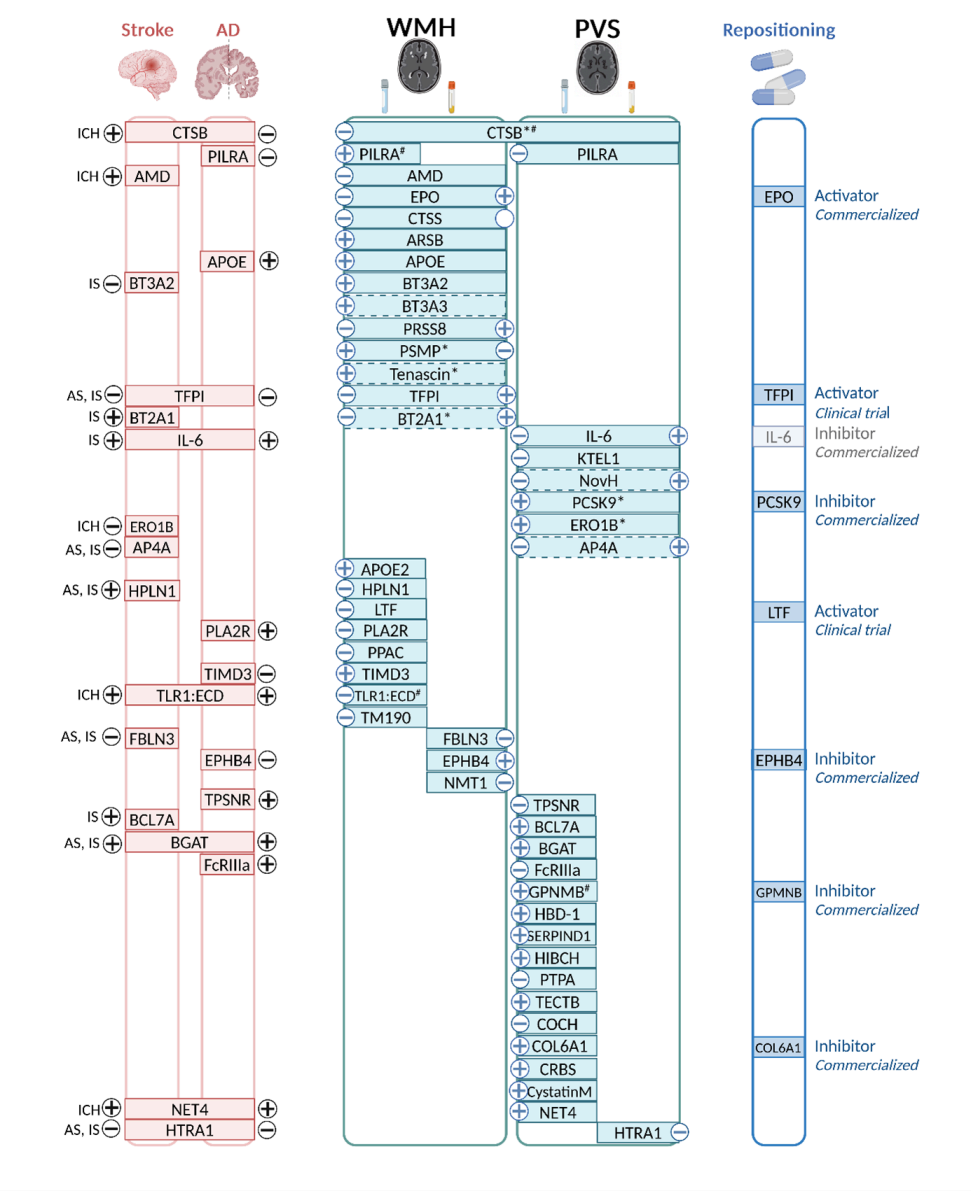
Integrated summary of our findings. Proteins associations with WMH, PVS, or both are represented in the middle. For each MRI-marker, the left side corresponds to CSF findings and the right side to plasma findings. * proteins with cross-ancestry association. ^#^ proteins with lifespan association. Associations with stroke Alzheimer’s disease (AD), or both are represented on the left of the figure. Subtypes of stroke are as follows: AS: Any stroke, IS: Ischemic stroke, SVS: Small vessel stroke, ICH: Intracerebral hemorrhage. − and + signs correspond to the direction of association referring to higher level of the protein. Drug repositioning is represented on the right of the figure.

Results of protein-cSVD associations along with clinical significance, pathway or cell-type enrichment and drug target identification are summarized in **Supplementary Table 18** and Fig. 6.

## Discussion

By combining a unique CSF and plasma pQTL resource with the latest GWAS of MRI-cSVD in a Mendelian randomization framework, we describe a new biological fingerprint of cSVD comprising 49 protein-cSVD associations with a putative causal relation, predominantly in the CSF. To assess robustness and specificity of our findings we implemented a multipronged follow-up approach, across fluids, proteomic platforms, and ancestries, which included testing associations of direct plasma protein measurements with MRI-cSVD. We highlight 16 proteins robustly associated in both CSF and plasma, of which 12 are in the same direction, while 24 and four proteins were identified in CSF or plasma only, with no evidence for association in the other fluid. Strikingly, several cSVD-associated proteins already showed associations with WMH and PVS burden at age 20 with consistent directionality. The fact that half of cSVD-associated proteins show at least nominally significant associations with stroke, AD, or both highlights their clinical relevance. Pathway and cell-type enrichment analyses suggest an important role of extracellular matrix and immune response pathways, with single-cell RNA-sequencing analyses pointing predominantly to microglia, but also oligodendrocytes, vascular smooth muscle cells and pericytes. Finally, besides revealing potential novel biomarkers and drug targets to be investigated, our findings also provide genetic support for repositioning of seven drugs for cSVD.

Previous explorations of cSVD proteomics were mainly conducted on focused protein panels^30,31^, mostly in plasma^14–17,32^ (except a recent study on 16 CSF proteins)^33^ and in relatively small cohorts (usually N < 1,000)^34^. Here we analyzed over 2,500 plasma and CSF proteins in relation with WMH and PVS burden in over 40,000 participants. In recent years, CSF biomarkers have emerged as pivotal for unraveling the intricate mechanisms underlying neurodegenerative and neuroinflammatory diseases, given their proximity to the central nervous system^35–37^. Our findings suggest that this also holds true for cSVD. Indeed, CSF-based MR analyses revealed five times more protein-cSVD associations than plasma-based MR, despite ten times smaller sample size to derive pQTL. Among proteins with pQTL available in both plasma and CSF resources, 67% of cSVD-associated plasma proteins also showed associations with the same MRI-cSVD markers in CSF, whereas only 24% of cSVD-associated CSF proteins showed associations in plasma. Even when accounting for follow-up with direct protein measurements, only 43% of cSVD-associated CSF proteins were associated with MRI-cSVD in plasma, suggesting that some protein-cSVD associations are specific to CSF, as described for other neurological disorders^12,13^.

Some proteins associated with MRI-cSVD were particularly robust, with consistent directionalities of their association across fluids and platforms, using both pQTL-based and direct measurements, especially, PILRA-deltaTM, PILRA-M14, ARSB and CTSB.

PILRA (paired immunoglobin like type 2 receptor alpha) is a microglial immunoreceptor involved in β-amyloid uptake and herpes simplex virus 1 infection^38^. Somascan measures soluble PILRA isoforms lacking the transmembrane domain^39^ (PILRA-deltaTM and PILRA-M14) while Olink detects the full protein. Higher genetically determined CSF levels of PILRA-M14 and PILRA-deltaTM were associated with larger WMH volume across the lifespan, notably already in young adults in their twenties. In contrast, higher genetically determined CSF and plasma levels of PILRA isoforms were associated with smaller WM-PVS burden and lower risk of AD (p < 10^− 23^ for high CSF levels). Higher plasma levels of PILRA (Olink direct measurements) were also protective for WM-PVS. This could potentially indirectly point to a protective effect of PILRA on cSVD caused by cerebral amyloid angiopathy (CAA), as WM-PVS was recently proposed as a novel CAA biomarker^40^, and CAA is associated with a strongly increased risk of AD^41^. Interestingly, previous experimental work has supported PILRA as the likely causal gene at the chr7q21 AD risk locus,^42^ suggesting that a common missense variant in this gene (rs1859788, r^2^ = 0.3 with PILRA pQTL) may protect against AD via reduced inhibitory signaling in microglia and reduced microglial infection during HSV-1 recurrence. The opposite effect we observed on WMH is intriguing, requiring further explorations, such as an examination of differential associations with WMH spatial patterns.

ARSB (arylsulfatase B) plays an important role in ECM degradation, regulation of neurite outgrowth and neuronal adaptability in the central nervous system^43^, where it is expressed predominantly in the microglia^44,45^. ARSB deficiency causes a lysosomal storage disorder (mucopolysacharidosisc)^46^. Here higher ARSB levels in CSF and plasma were associated with greater WMH volume based on both Somascan pQTL and direct Olink protein measurements, making ARSB a compelling candidate to explore as a circulating cSVD biomarker. CTSB (cathepsin B) is a cerebrovascular matrisome-associated protein identified in brain microvessels^31^. This lysosomal cysteine protease is involved in proteolysis of ECM components and enhanced vessel wall permeability^47^, as well as in proteolysis of amyloid precursor protein, implicated in AD^48^. Genetically determined higher CTSB levels in CSF were associated with smaller WMH and BG-PVS burden, replicating in plasma, across platforms (pQTL and direct measurements) and ancestries, and with lower AD risk at nominal significance. Similar associations were observed between higher genetically determined CSF and plasma levels of CTSS (cathepsin S, another cysteine protease) and smaller WMH volume, but higher direct plasma CTSS measurements were associated with larger WMH volume. A potential explanation for such discrepancies could be that pQTL and direct measurements capture different isoforms (Olink assays have been developed for the canonical CTSS isoform 1). Noteworthy, rare mutations in CTSA (encoding cathepsin A, a serine protease like HTRA1) cause a rare monogenic autosomal recessive cSVD known as CARASAL^49^. Our findings thus expand the involvement of cathepsins to complex cSVD, and to cysteine in addition to serine proteases.

We also show for the first time an association of lower plasma levels of HTRA1 (High-Temperature Requirement A serine peptidase 1), another cerebrovascular matrisome protein, with extensive HIP-PVS, consistent with loss-of-function mechanisms underlying monogenic cSVD caused by rare mutations in *HTRA1* (CARASIL, autosomal dominant *HTRA1* mutations)^50^. Rare and common variants at *HTRA1* have been associated with larger WMH volume and increased stroke risk in the general population^51,52^, with recent findings suggesting loss-of-function mechanisms through both reduced HTRA1 expression and lower serine protease enzyme activity. The association of lower genetically determined plasma levels of HTRA1 with extensive HIP-PVS provides additional evidence for an impact of HTRA1 loss-of-function on brain health. Interestingly, lower genetically determined HTRA1 plasma protein levels were also associated with higher risk of stroke (any, ischemic) and AD at p < 0.05.

Overall, our proteogenomic analyses lend support to a prominent role of the cerebrovascular matrisome (extracellular matrix and associated proteins) in both monogenic and multifactorial cSVD, corroborating and expanding findings from large genomic studies^5,6^ and preclinical work on monogenic cSVD models^31^. In parallel, our findings also reveal prominent associations of immune response pathways with MRI-cSVD. Intriguingly, associations with proteins involved in immunity and inflammation (with PILRA, TLR1, GPNMB, all three expressed predominantly in microglia) were already detectable in young adults in their twenties. We also found expression of genes encoding CSF cSVD-associated proteins to be significantly enriched in microglial cells, the brain’s primary resident immune cells. The interplay between cSVD and inflammation has gained recent interest, with emerging evidence from focused biomarker studies and experimental models, suggesting that activation of immune cells and in particular microglial cells could play an important role^53–57^. Co-registration of MRI images with (immune-)histopathological data has shown that WMH volume was associated with higher microglial activation, supporting that the latter could be involved in cSVD etiology^58^. Our results lend further support to this, suggesting that this could be one of the earliest processes involved, as demonstrated for AD^59^. Given growing evidence that changes in microglial transcriptional profiles play a crucial role in brain aging and AD and that blood proteins can mediate neurotoxic microglial functions^60^, the proteogenomic signature we describe might contribute to revealing biological underpinnings of the intricate relation between cSVD and AD^29,61,62^.

Some cSVD-associated proteins are encoded by genes in cSVD GWAS loci, strengthening evidence for their involvement in disease pathogenesis. At chr17q21, lower plasma levels of NMT1 (N-Myristoyltransferase1), a protein involved in vascular instability and endothelial cell damage^63–65^, were associated with larger WMH volume, aligning with prior associations of lower arterial *NMT1* expression with larger WMH burden^66^. At chr2p16, lower plasma levels of FBLN3 (Fibulin-3, encoded by *EFEMP1*), a glycoprotein essential for maintaining ECM and vessel integrity and involved in cell proliferation and migration, were associated with larger WMH volume^23,67^. Furthermore, beyond genetic risk scores derived from cSVD GWAS, genetic risk scores for cSVD-associated proteins may have added predictive value for identifying those with extensive cSVD burden, highlighting the potential of multiomics approaches for enhancing risk prediction and stratification.

This work further unveiled new prospects for therapeutic repositioning and development, with the identification of seven drugs (targeting EPO, LTF, TFPI, EphB4, COL6A1, GPNMB, and PCSK9) with cSVD MR results compatible with potential beneficial therapeutic effects, warranting further investigation. Of these, agonists for EPO and LTF and inhibitors of EphB4, which are either approved or studied in phase II clinical trials for other indications (**Supplementary Table 28**) present evidence of successfully crossing the blood brain barrier (BBB), although it is unclear whether this is required to treat cSVD. EPO is a neuroprotective protein safeguarding the BBB against VEGF-induced permeability^68^, acting through the Keap1/Nrf2 pathway in ischemia reperfusion injury^69^. LTF has anti-inflammatory and neuroprotective properties and can upregulate EPO^69^ and downregulate IL-6^70,71^, both associated with MRI-cSVD in our study. EPO and LTF were reported to show strong protein-protein interaction with collaborative anti-inflammatory properties^69^ and modified, optimized versions of both these proteins have been tested experimentally as neuroprotective agents in ischemic stroke and intracerebral hemorrhage, and, for some, patented (WO2006120030A1)^72–74^. Erythropoietin-producing hepatocellular receptor B4 (EphB4), a tyrosine kinase receptor expressed in vascular endothelial cells, plays a crucial role in vascular development and adult vascular biology, influencing blood vessel permeability, inflammation, and angiogenesis through interaction with the Notch pathway^75^. Drugs inhibiting PCSK9, COL6A1, or GPNMB and enhancing TFPI may hold promise for cSVD as well (**Supplementary Table 28)**. PCSK9 is a convertase strongly linked to lipid homeostasis but also involved in neuronal apoptosis, neurogenesis, and brain inflammation^76^. Elevated PCSK9 levels have been associated with ischemic stroke (plasma) and AD (CSF)^76^. A protective effect of PCSK9 inhibitors on ischemic stroke has been demonstrated^77^. More recently, PCSK9 was shown to regulate amyloid beta clearance from the brain and peripheral PCSK9 inhibition reduced Aβ pathology in prefrontal cortex and hippocampus in mice^78^. Here, the robust association of high PCSK9 levels with larger WM-PVS burden, both in Europeans (CSF, Somascan pQTL) and East-Asians (plasma Somascan direct measurements), could suggest an association with the CAA subtype of cSVD^40^, characterized by Aβ deposition in the brain vasculature. The bi-directional MR result suggesting not only a putative causal association of higher PCSK9 levels with WM-PVS, but also an association of larger genetically determined WM-PVS burden with higher CSF PSCK9 levels is intriguing. Extensive WM-PVS burden is believed to reflect underlying glymphatic dysfunction, involved in impaired clearance of amyloid beta, but also other substances from the brain^79^.

Strengths of our study include the large-scale proteogenomics approach in plasma and CSF, using a Mendelian randomization framework that provides evidence for potential causality. The multipronged follow-up strategy across fluids and platforms strongly enhances the robustness of our findings. Although limited by smaller sample size, the extension across the lifespan and to East-Asian ancestry groups is unique and provides crucial insights on early life mechanisms underlying cSVD, while enabling transportability of findings to East-Asian populations where cSVD is particularly prevalent^80^. We acknowledge limitations. pQTL were derived from a population enriched in neurologically impaired individuals (especially AD patients), however we previously showed that pQTL are only marginally influenced by disease status^12^; moreover, follow-up samples were not enriched in AD patients. Although we have used the largest available commercial panel, discovery was limited to proteins quantified by Somascan, for which valid pQTL instruments could be derived, representing less than 10% of known proteins (without accounting for isoforms). We had no available sample for following up associations in the CSF, given the scarcity of CSF proteomics resources, and the fact that lumbar puncture is typically not done in the context of cSVD. Non-significant follow-up of associations discovered using Somascan pQTL with Olink direct plasma protein measurements may reflect spurious findings but also lack of power or modest correlation across platforms due to distinct technology. Inconsistent directionality of some significant associations between pQTL analyses and direct measurements or between both platforms requires further exploration but could reflect that distinct isoforms are being captured. Overall, these complexities highlight the importance of multiple follow-up and validation steps when interpreting association results from high-throughput proteomics assays.

## Conclusion

Our work provides an extensive, first *in vivo* biological fingerprint of cSVD derived from large-scale proteogenomics studies in CSF and blood. The results highlight important biological processes underlying cSVD at the molecular and cellular levels, pointing to shared pathways between cSVD and AD of potential therapeutic relevance and early life mechanisms involving immunity and inflammation. This proteogenomic signature paves the way for deriving circulating biomarkers and exploring drug development and repositioning opportunities.

## Methods

### Discovery of protein-cSVD associations

We applied two-sample Mendelian randomization (MR) analyses to explore the relation of genetically predicted cerebrospinal fluid (CSF) and plasma protein levels with MRI-markers of cerebral small vessel disease (MRI-cSVD).

#### Deriving genetic instruments for circulating protein levels (instrumental variables for the exposure) using protein quantitative trait loci (pQTL)

pQTL were generated from genome-wide association studies (GWAS) of circulating protein levels. CSF pQTL summary statistics were obtained from 7,028 proteins measured on the aptamer-based Somascan 7k platform in 3,107 research participants of European ancestry. Of these, 1,076 participants were cognitively normal, 1,001 had clinically determined late-onset Alzheimer’s disease (AD), 118 had early-onset AD, 281 non-AD dementia, and 631 Parkinson’s disease.^12^ Plasma pQTL summary statistics were obtained from 4,907 proteins measured on the Somascan 5k platform in 35,559 cognitively normal individuals of European ancestry^19^ participating in either the Icelandic cancer project (52%) or deCODE genetics (48%). Cis-pQTL were defined as genetic variants within 1 Mb of the corresponding protein coding gene. Genetic variants were selected based on genome-wide significant associations (p<5×10^−8^) with protein abundance after clumping using PLINK2^81^ for linkage disequilibrium at r^2^<0.01, within 1 Mb. Genetic variants included in the MHC region (chr6:26Mb-34Mb) were removed considering the complex LD structure of the region. The strength of the instrumental variables (IV) was measured using the F-statistic (instruments with an F-statistic > 10 were considered strong). Following these steps, we selected up to 1,121 CSF and 1,732 plasma proteins with cis-acting pQTLs; as well as 2,805 CSF and 4,614 plasma proteins with cis- and trans-acting pQTLs for MR analyses.

#### Genetic associations with MRI-cSVD (outcome)

We used summary statistics from the latest, largest GWAS meta-analyses of white matter hyperintensity (WMH) volume, in 48,454 participants (mean age 66.0 years), and of extensive perivascular space burden (PVS) in white matter (WM), basal ganglia (BG) and hippocampus (HIP), in 38,525 participants (mean age 68.3 years), from the general population, of European ancestry, and free of stroke (described in detail elsewhere^5,20^). Importantly, the cohorts from which the pQTL were derived were not included in these WMH and PVS GWAS meta-analyses.

#### Analytical steps for Mendelian randomization analyses

MR analysis was performed using R version 4.1.0, the “TwoSampleMR” package version 0.5.6^82^. We applied two-sample MR analyses to assess the causal association between genetically predicted CSF and plasma protein levels and MRI-cSVD. pQTL obtained after instrument selection for each protein were used as instrumental variables (IVs). We extracted the association estimates between the variants and the exposures or the outcomes and aligned the effect alleles using the *TwoSampleMR* R package.

For proteins with multiple IVs we computed MR estimates with random-effect Inverse Variance Weighted (IVW) analysis^83^ that rely on distinct assumptions for validity: (i) Heterogeneity across the MR estimates was assessed for each instrument using Cochran’s Q statistic (p<0.05 was considered significant)^83^; (ii) Horizontal pleiotropy was assessed using MR-Egger intercept as a measure of directional pleiotropy (p<0.05 was considered significant)^84^. We further conducted various sensitivity analyses^85^:

1. The identification of outlier IVs and their removal from analyses was conducted using MR Pleiotropy residual Sum and Outlier (MR-PRESSO)^86^ (p<0.05 was considered significant)
2. Reverse MR was run by reversing the direction of inference, using the MRI-cSVD markers as the exposure and proteins as the outcome, to formally rule out reverse causation.
3. MR-Egger regression^87^ and Weighted median that are more robust to the use of pleiotropic instruments were used as sensitivity analyses. When pleiotropy was observed, we retained results when at least 2 of the 3 sensitivity methods (MR-Egger, Weighted median, MR-PRESSO) were concordant with each other and p<0.05.

For proteins with a single IV we computed MR estimates using the Wald ratio. MR analyses were followed by colocalization analyses using coloc^88^ including variants ±1Mb surrounding the pQTL of interest. Associations were considered significant when the posterior probability H4 (PPH4; shared association with single causal variant) was ≥0.70 and suggestive for PPH4<0.70 when posterior probability H3 (PPH3; shared association with different causal variant)<0.70^89^. Associations with PPH4<0.70 and PPH3 >0.70 were removed from further analyses.

Discovery MR results were considered significant when passing the FDR Benjamini-Hochberg corrected significance threshold (P_FDR_<0.05). In sensitivity analyses we additionally corrected for the number of independent phenotypes tested, estimated using correlations between traits in the 3C study applying the Matrix Spectral Decomposition (matSpDlite^90^) method for WMH volume and each PVS location, (p_FDR_<1.2×10^−2^; 0.05/4).

#### Genetic correlation of identified protein-cSVD

Genetic correlations were performed using LDSC to identify proteins that may have a shared genetic basis leveraging pQTL summary statistics of the 45 proteins identified in CSF and 9 proteins identified in plasma. Only proteins with heritability greater than 20% could be used (N_CSF_=24, N_plasma_=9). (p<5×10^−5^ was used correcting for the mean of proteins tested and 3 situations: CSF-CSF, CSF-plasma and plasma-plasma; 0.05/18*18*3)

### Follow-up of significant protein-cSVD associations

We used a multi-pronged approach to follow-up significant protein-cSVD associations, across fluids, platforms, and ancestries.

#### Cross-fluid follow-up (pQTL, Somascan, plasma and CSF)

Proteins identified in one fluid were followed up for association with MRI-cSVD in the other fluid. Out of CSF or plasma cSVD-associated proteins, we selected proteins for which genetic instruments were available in both datasets^12,19^ (N=43). Significant associations were defined by p_FDR_<0.05. In addition, results of sensitivity analyses at p_FDR_<1.2×10^−2^ are displayed, accounting for the 4 phenotypes tested.

#### Cross-platform follow-up (direct protein measurements, Olink, plasma)

Two large population-based cohort studies were used to follow-up protein-cSVD associations in participants with both MRI-cSVD phenotypes and plasma proteomic measurements, using paired nucleotide-labeled antibody probes (Olink Explore 3072): 3C-Dijon (WMH, BG-PVS, WM-PVS and HIP-PVS) and UK Biobank (WMH, BG-PVS and WM-PVS).

The 3C-Dijon study is a population-based cohort study comprising 4,931 participants aged 65 years and older at inclusion recruited between 1999 and 2001^91,92^. A subset of 1,924 participants aged <80 years took part in an ancillary brain imaging study (1.5T Siemens Magneton scanner). Olink proteomic profiling, based on blood samples obtained at inclusion, was conducted in 1,056 participants selected based on availability of brain MRI and amounts of plasma tubes left (**Supplementary Table 12)**. Protein measurements were conducted on the Olink Explore 3072 panel using Proximity Extension Assay (PEA) technology, following the manufacturer’s protocol^93^, at McGill Genome Center (Montreal, Canada). This panel measures 2,941 protein analytes and captures 2,923 unique proteins across 8 protein panels (cardiometabolic, cardiometabolic II, inflammation, inflammation II, neurology, neurology II, oncology and oncology II)^94^. Data pre-processing including plate-based normalization and QC checks were conducted according to standardized Olink protocols. WMH volume was estimated using a multimodal (T1, T2, DP) image processing algorithm^92.^ PVS burden in basal ganglia and white matter was estimated with the previously described machine-learning based SHIVA-PVS algorithm^1,2^ using T1-weighted images; while PVS burden in hippocampus was estimated using a previously described visual semi-quantitative rating scale^95^.

The UK Biobank (UKB) study is a British cohort following 502,620 participants recruited between 2006 and 2010. Proteomic profiling was performed on plasma samples collected at baseline from 54,219 UKB participants using Olink Explore 3072 (field ID: 1839), with QC conducted following the protocol implemented by UKB (resource 4658). Of these, 5,494 also underwent a brain MRI (3T Siemens Prisma scanner), with WMH volume measurements (field ID: 25008), and were used for analysis. PVS burden (in basal ganglia and white matter) was estimated with the previously described machine-learning based SHIVA-PVS algorithm^96,97^ using T1-weighted images from the subset of 5,523 participants with proteomics data (**Supplementary Methods**).

We conducted multivariable linear and logistic regression of individual proteins with WMH volume and PVS burden adjusted for the delay between age at blood draw and age at the time of MRI, sex, batch effect, total intracranial volume (or mask volume for WMH in 3C-Dijon). WMH volume and PVS burden in basal ganglia and white matter were inverse normal transformed and PVS in hippocampus values were dichotomized, comparing participants in the top quartile of PVS burden distribution to the rest, as previously described^5^. An inverse variance weighted meta-analysis was performed using *metafor* R package^98^ to combine 3C-Dijon and UKB association analyses. The heterogeneity of associations across studies was assessed using the Cochran-Mantel-Haenszel statistical test, only associations with p>1.9×10^−3^ (0.05/26, correcting for 26 proteins available for follow-up) were considered. Significant associations were defined by p_FDR_<0.05. In addition, results of sensitivity analyses at p_FDR_<1.2×10^−2^ are displayed, accounting for the 4 phenotypes tested.

Correlation analyses between protein levels were conducted in UKB (the largest of the two samples) using the *corrplot*^99^ R package. Correlations were defined as significant at the Bonferroni corrected p-value threshold of p<7.7×10^−5^ (0.05/(26*26)-26).

#### Cross-ancestry follow-up (direct protein measurements, Somascan, plasma)

Brain imaging and plasma proteomic data from the Nagahama study, a prospective population-based cohort study initiated in 2007 in Nagahama, Japan (N=10,082 at baseline) were used^100^. Healthy participants (without serious physical impairment and heath issue) aged 30 to 74 years were recruited between 2008 and 2010 from the general population of Nagahama (Japan) and followed-up 5 years after baseline between 2013 and 2015. Plasma proteomic measurements have been conducted on a subset of 2,000 individuals using Somascan 4.0. Of those, 858 had brain MRI measurements. WMH in Nagahama was generated using UBO detector^101^, a publicly available automated tool which extracts features from T1w and FLAIR input images, such as relative intensity levels, tissue probability, and anatomical location, to classify FLAIR hyperintensities as WMH using k-Nearest Neighbor algorithm. A trained rater reviewed visual quality control report generated by the tool to reject gross failures in tissue probability estimates and WMH classification. PVS burden was estimated using the aforementioned machine-learning based SHIVA-PVS algorithm^5,96^. QC checks and proteomic measurements transformation (log2) were conducted according to standardized Somascan protocols. After excluding participants for whom the estimation of the MRI-marker was not possible, without proteomics measurements passing QC, with prevalent stroke, missing covariates, or who had withdrawn their consent, a total of 785 participants were available for association analyses. We conducted linear regression for WMH, WM-PVS and BG-PVS as continuous variables inverse normal transformed adjusted for age, sex, batch, total intracranial volume and the first 4 principal components. Significant associations at p_FDR_<0.05 were reported. Given the exploratory nature of these cross-ancestry analyses on a much small sample size, associations at p<0.05 were also reported.

#### Follow-up across the lifespan (pQTL, Somascan, plasma and CSF)

We conducted two-sample MR analyses using the aforementioned pQTL in plasma and CSF (instruments) and GWAS for WMH and PVS (outcomes). WMH and PVS GWAS were conducted in the Internet-based Students HeAlth Research Enterprise (i-Share) study, an ongoing prospective population-based cohort study of French-speaking students^102^. We specifically leveraged the bio-Share ancillary study, a biological platform comprising a collection of blood samples from a subset of the i-Share cohort, and MRi-Share, an ancillary study comprising a brain MRI (3 Tesla Siemens Prisma scanner) and a battery of cognitive tests^103–105^. Here we used the sub-sample of 1,748 MRi-Share and bio-Share participants aged 18–35 years, recruited in Bordeaux, France, for whom both brain MRI and genome-wide genotype data were available (mean age ± standard deviation (SD): 22.1±2.3 years; 72.2% women)^105^. MRI protocol, genetic data quality control and imputation procedures are detailed elsewhere^5,103–105^. For i-Share PVS GWAS summary statistics, we used previously described data^5^. For i-Share WMH GWAS summary statistics, we performed GWAS using the genome-wide linear mixed model implemented in REGENIE on WMH volume quantified using a recently developed algorithm^106^ (after excluding 8 participants with multiple sclerosis or radiologically isolated syndrome)^107^. WMH volume was transformed using an indirect inverse normal transformation (applying inverse normal transformation to residuals from linear regression of WMH adjusted for covariates [age at MRI, sex, total intracranial volume, and the first four principal components of population stratification]). These analyses were restricted to SNPs with an imputation score >0.5 and a MAF>0.01 and were adjusted for age at MRI, sex, intracranial volume and the first four principal components of population stratification.

Following the steps of instrument selection and MR previously described, we performed two-sample MR between each of the 49 proteins associated with MRI-cSVD using the large meta-analyses in older adults and MRI-cSVD measured in young adults. Associations were defined as nominally significant if p<0.05, and significant when p_FDR_<0.05.

Significant associations at p_FDR_<0.05 were reported. Given the exploratory nature of these lifespan analyses on a much small sample size, associations at p<0.05 were also reported.

### Protein genetic risk scores (protein-GRS)

Quality control of genotypes and summary statistics are detailed in the Supplementary Methods.

#### Construction of GRS

We constructed GRS for each of the 49 cSVD-associated proteins that passed sensitivity analyses using a standard clumping and thresholding approach^100,108^. We used *PRSice-2* software to clump SNPs with r^2^<0.1 using the 1000G European subset as a reference panel, and select SNPs from cis-pQTLs reaching genome-wide significance (p<5×10^−8^)^109^. A GRS for each protein was derived using the standard weighting method:

$$GRS_{i}=\sum_{j=1}^{m}\;x_{ij}{\overset{ˆ}{\beta }}_{j}$$

where $x_{ij}\in \{0,1,2\}$ is the count of risk alleles for the j-th SNP for the i-th individual, and ${\overset{ˆ}{\beta }}_{j}$ is the effect size for the j-th variant in the pQTL summary statistics.

#### Association analysis with extreme-cSVD

We investigated the ability of protein-GRS to predict extremes of cSVD severity (extreme-cSVD) in the 3C-Dijon cohort^92^. Briefly, after removing individuals with prevalent stroke, dementia, or brain tumor, we defined a binary phenotype for extreme-cSVD in 1,497 participants with MRI and genome-wide genotype data (N=58 extensive, with WMH burden in the top quartile of the cohort distribution ± presence of lacunes; 253 minimal-cSVD, with WMH burden in the bottom quartile of the cohort distribution and no lacunes or other types of brain infarcts, **Supplementary methods**).

We performed logistic regression of each of the standardized protein-GRS with extreme-cSVD as the dependent variable, adjusting for the first 5 principal components for population stratification^110^. We also used a previously derived WMH GRS (weighted sum of independent genome-wide significant risk variants for WMH volume), a strong genetic predictor of WMH volume, for comparison^20^. The number of SNPs in each GRS is included in **Supplementary Table 19.** We found five genetically determined CSF and plasma proteins nominally associated with extreme-cSVD, although none remained significant after Bonferroni-correction for the 49 protein-GRS (p<0.001).

#### Prediction Performance

We assessed the performance of these 5 protein-GRS to predict extreme-cSVD, individually and combined, in models adjusted for the first 5 principal components and WMH-GRS: *CSF.Cystatin-M, PLASMA.PILRA-M14, CSF.PPAC, PLASMA.PILRA-deltaTM*, and *CSF.TLR1-ECD*. As PILRA isoforms were extremely correlated (r=0.99), we selected the isoform displaying the strongest association with extreme cSVD (PILRA-M14) for the combined model. Prediction performance was evaluated in 3C-Dijon through internally validated AUC using the optimism bootstrap estimator in the *caret* R package (2,000 bootstrap replications)^111^.

### Clinical significance

To explore the relation of genetically determined protein levels with clinical complications of cSVD, we used summary statistics of the largest available GWAS (European ancestry subset) for stroke and dementia. Summary statistics for any stroke, ischemic stroke, and small vessel stroke were derived from the GIGASTROKE study (comprising 73,652 patients with any stroke, 62,100 with ischemic stroke, and 6,811 with small vessel stroke^112^) and the largest publicly available GWAS for intracerebral hemorrhage (ICH, 1,545 patients^113^). For dementia we used summary statistics of the largest GWAS for Alzheimer’s disease comprising 71,880 AD cases^114^.

Following the steps of instrument selection and MR described above, we performed two-sample MR to test the relation of each genetically determined levels (in plasma and CSF) of the 49 cSVD associated proteins with stroke (subtypes) and dementia. To capture trends towards clinical significance we considered associations at p<0.05 and reported significant findings after multiple testing correction at p_FDR_<0.05.

#### Cross-ancestry

To assess the causal association between serum protein levels of cSVD-associated proteins and stroke, in individuals of East-Asian ancestry, we conducted two-sample MR analyses in BioBank Japan (BBJ, first cohort study^115^), which recruited around 200,000 participants with at least one of 47 target diseases across 66 hospitals in Japan between 2003 and 2007.. Proteomic profiling was conducted for a total of 2,886 individuals of East-Asian ancestry from two previous studies^116,117^ with whole genome sequencing datasets, using the Olink Explore 3072 panel following the manufacturer’s protocol. Data pre-processing, including intensity normalization, bridge normalization across batches, and QC, was conducted according to standardized Olink protocols. Rank-based inverse normal transformation was applied to protein level measurements before association tests. pQTL summary statistics of serum protein levels were obtained for 19 available proteins (out of the 49 cSVD-associated proteins from the discovery analysis) by meta-analyzing summary statistics generated in individuals from each study separately using REGENIE v3.2.9^107^ (adjusted for age, sex, age^2^,age*sex, age^2^*sex, batch, and the first 10 genotype principal components) and METAL^118^ (inverse variance weighted method; fixed effect model). Summary statistics of GWAS for ischemic stroke (N=17,493), large-artery atherosclerotic stroke (N=1,322), cardioembolic stroke (N=747), and small vessel stroke (N=4,876) were obtained in the BBJ first cohort using REGENIE v3.2.9 (adjusted for age, sex, and the first 10 genotype principal components), excluding the samples used for proteomic profiling. Genotyping, quality control, and imputation for BBJ samples used in the stroke GWASs were conducted as previously described^119^, except that the imputation was performed using a reference panel combining the 1000 Genome Project phase 3 v5 reference panel and 3256 Japanese samples (JEWEL3k) samples^120^. Individuals without any type of stroke or cerebral aneurysm were used as controls. Instrument selection and MR were conducted following the methods previously described (p-threshold for clumping: 1×10^−6^, **Supplementary methods**)

### Biological interpretation

#### Protein-protein interactions

Protein-protein interactions were analyzed using the STRING database with the initial set of 1,121 proteins for CSF and 2,805 for plasma as background.

#### Pathway enrichment analysis

The GENE2FUNC analysis tool in FUMA (v1.5.4) was employed to conduct gene set enrichment analyses and detect significantly associated GO biological processes^121^. GENE2FUNC employs a hypergeometric test to assess the over-representation of genes within predefined gene sets, including GO biological processes. The gene IDs used correspond to coding-genes of identified proteins. We tested enrichment of the entire set of genes encoding cSVD-associated proteins identified in CSF and plasma, using the background set of genes encoding proteins tested for MR in each tissue respectively (**Supplementary Table 1**). Benjamini-Hochberg multiple testing correction was applied to these results (p<0.05).

#### STEAP enrichment analysis

We performed a cell type enrichment analysis using the Single cell Type Enrichment Analysis for Phenotypes (STEAP) tool (https://github.com/erwinerdem/STEAP/). This tool serves as an extension to CELLECT and integrates stratified LD-score regression (S-LDSC), MAGMA, and H-MAGMA for enrichment analysis. pQTLs summary statistics from the CSF and plasma datasets were preprocessed. Subsequently, expression specificity profiles were computed using single-cell RNA sequencing data from human and mouse databases, including PsychENCODE DER-22, GSE67835, GSE101601, DroNc Human Hippocampus, Allen Brain Atlas MTG and LNG, Mousebrain, Tabula Muris, Descartes Human Cerebrum, and Cerebellum. Cell type enrichment analysis was conducted employing MAGMA, H-MAGMA (which incorporates chromatin interaction profiles from human brain tissues in MAGMA), and S-LDSC. P-values were Bonferroni corrected for the number of independent cell types in each database.

#### Brain single cell expression quantitative trait loci (eQTL)

Mapping of brain single cell eQTL was described elsewhere^26^. Briefly, single-nucleus RNA-seq libraries were prepared from dorsolateral prefrontal cortex (dPFC) of 424 participants from the ROSMAP cohort using 10x Genomics Single Cell 3’ kit. Sequencing reads were processed and UMI count matrix was generated using Cell Ranger software (ver.6.0.0, 10x Genomics). Classification of cell types were performed by clustering cells by gene expression using the R package Seurat (ver. 4). “Pseudobulk” gene expression matrix was constructed by aggregating UMI counts of the same cell type of the same donor and normalizing them to the log2 counts per million reads mapped (CPM) values. Genotyping was performed by whole genome sequencing and GATK. Mapping of cis-eQTL was performed using Matrix-eQTL (ver. 2.3) for SNP within 1 Mb from transcription start sites.

Due to the sparsity of vascular cells in brain tissue, a specific dataset from ROSMAP using *in silico* vasculature enrichment was used for eQTL and expression analysis. Single-nucleus RNA-seq libraries were prepared from brain samples of 409 ROSMAP participants using the 10x Genomics Single Cell 3′ Kit. Read counts were estimated using Cellranger 3.0.1 (10x Genomics) and the UMI count matrix was analysed using the Seurat R package v.3.2.0. Vascular enrichment was conducted *in silico* using cell sorting from post mortem human samples across seven different brain regions (prefrontal cortex, mid-temporal cortex, angular gyrus, entorhinal cortex, thalamus, hippocampus and mammillary body). Cell-type annotation was performed through clustering, annotating cell-type using a combination of canonical vasculature markers and whole-transcriptome cellular signatures. Detailed methods regarding sRNAseq and *in silico* vascular enrichment is described elsewhere^27,28^. Microglia states were defined from 152,459 microglial transcriptomes across 443 individuals (217 AD and 226 controls) identifying 12 transcriptional states. Microglial nuclei were obtained from post-mortem brain samples from the ROSMAP study across 6 brain regions (hippocampus, dPFC, mid-temporal cortex, angular gyrus, entorhinal cortex and thalamus). Using in silico sorting, 174,420 immune cells were collected from snRNA-seq datasets using STAR method forming 12 clusters of microglia. Those clusters were then defined as microglia states based on their molecular signature and function: MG0: hemostatic, MG1: neuronal surveillance, MG2: Inflammatory I, MG3: Ribosome biogenesis, MG4: Lipid Processing, MG5: Phagocytic, MG6: Stress signature, MG7: Glycolytic; MG8: Inflammatory II, MG10: Inflammatory III, MG11: Antiviral, MG12: Cycling. Detailed methods regarding microglial states definition are described elsewhere^29^.

### Proteomics driven drug discovery

Using significant MR results from CSF and plasma, we restricted our analysis to drug-targeting proteins using 4 drug-gene databases (ChEMBL, pharmGKB, DrugBank and TTD). Following this methodology, eight drug-targeting proteins were identified for WMH (EPO, LTF, TFPI, APOE, ARSB, CTSS, CTSB and EPHB4) and seven for PVS (COL6A1, CTSB, GPNMB, PCSK9, FcRIIIA, Heparin co-factor II, IL6). Using public drug databases, we then curated drugs targeting those proteins in a direction compatible with a beneficial therapeutic effect against the corresponding cSVD phenotype based on MR estimates. The desired mode of action (MoA) was defined as the opposite direction of the MR estimate. Once the drugs were identified, we searched the literature for a potential action of the drug.

## Data Availability

We used publicly available data for analyses described in this manuscript, including data from GWAS catalog (https://www.ebi.ac.uk/gwas/, study code: GCST90244151, GCST011947, GCST007320, GCST90104539, GCST90162546), the DECODE project (https://www.decode.com/summarydata/), ChEMBL (https://www.ebi.ac.uk/chembl/), pharmGKB (https://www.pharmgkb.org/), DrugBank (https://go.drugbank.com/), TTD (https://db.idrblab.net/ttd/), CSF pQTL summary statistics available at NIAGADS (full summary statistics available to approved investigators through accession #NG00130), NeuroGenomics and Informatics Center website (https://neurogenomics.wustl.edu/open-science/raw-data/) and ONTIME browser (https://ontime.wustl.edu/).
